# Congenital Cytomegalovirus Infection: From Silent Threat to Vaccine Horizon

**DOI:** 10.3390/vaccines13090929

**Published:** 2025-08-30

**Authors:** Rafaela Anna Moutsopoulou, Aikaterini Markou, Alexandra Lianou, Konstantina Leontari, Zoi Iliodromiti, Theodora Boutsikou, Georgios Kafalidis, Styliani Paliatsiou, Paraskevi Volaki, Nicoletta Iacovidou, Andreas G. Tsantes, Rozeta Sokou

**Affiliations:** 1 Neonatal Department, Aretaieio Hospital, School of Medicine, National and Kapodistrian University of Athens, 11528 Athens, Greece; moutsopoulourafaela@gmail.com (R.A.M.); cathymarkou@gmail.com (A.M.); kleontari@yahoo.gr (K.L.); ziliodromiti@yahoo.gr (Z.I.); theobtsk@gmail.com (T.B.); gkafalidis@gmail.com (G.K.); stpaliatsiou@yahoo.gr (S.P.); v.volaki@hotmail.com (P.V.); niciac58@gmail.com (N.I.); 2 Neonatal Intensive Care Unit, General Hospital of Nikaia “Agios Panteleimon”, 18454 Piraeus, Greece; alexlianou95@gmail.com; 3 Microbiology Department, “Saint Savvas” Oncology Hospital, 11522 Athens, Greece

**Keywords:** congenital cytomegalovirus infection, immunology and infectious diseases, neonates, vaccine, congenital infections, prevention strategy

## Abstract

Congenital cytomegalovirus (cCMV) infection is the most prevalent congenital infection, affecting approximately 0.5–2% of newborns, and is the leading non-genetic cause of sensorineural hearing loss and neurological impairment. The most severe outcome occurs following primary maternal infection during the first trimester of pregnancy, and up to 40–50% of affected fetuses sustain permanent damage. Diagnosis relies on early prenatal screening through maternal serum testing, optimally performed in the first trimester, followed by confirmatory amniocentesis after 17 weeks’ gestation. Prenatal imaging with ultrasound and magnetic resonance imaging (MRI) plays a critical role in the identification of fetal brain abnormalities. Prevention strategies emphasize hygiene measures aimed at reducing maternal exposure to bodily fluids of young children, particularly prior to conception and during early pregnancy. Despite progress in vaccine development, currently available ones demonstrate modest efficacy. This review presents a comprehensive summary of congenital CMV infection, addressing its epidemiology, pathogenesis, diagnostic approaches, clinical presentation, and preventive measures, with a focus on recent advances in vaccine research.

## 1. Introduction

Congenital cytomegalovirus infection (cCMV), caused exclusively by Human cytomegalovirus (HCMV), is the most common viral congenital infection worldwide affecting 0.5–2% of live births, and represents the leading non-genetic cause of sensorineural hearing loss (SNHL) and neurological impairment [1]. HCMV is a double stranded DNA beta herpesvirus (human herpesvirus-5 (HHV-5)) [2] that exhibits high species specificity, with humans being its exclusive natural host. It is ubiquitous, endemic worldwide and shows no seasonal pattern in its occurrence [3]. As with other herpesviruses, it establishes a lifelong infection, by settling latently after the initial infection [4]. Subsequently, the virus may occasionally be reactivated or a recurrent infection by different CMV strains that circulate throughout the world may occur [5,6]. CMV can be transmitted from a pregnant mother to her fetus through the placenta, causing congenital infection. The clinical presentation of cCMV infection varies widely, ranging from no apparent symptoms to severe complications. Notably, CMV is one of the primary causes of S and neurodevelopmental disabilities that are not inherited genetically in children [7]. It is estimated that approximately 10–20% of congenitally infected newborns will develop permanent impairments, such as hearing and vision loss or neurological disabilities, including cerebral palsy, cognitive impairment, and seizures [7,8]. The risk for severe complications is particularly high following maternal primary infection (MPI) during the first trimester, and up to 40–50% of affected offsprings develop permanent sequelae [8]. Preventive strategies include minimizing parental exposure to body fluids of young children, especially prior to conception and during early pregnancy. Despite ongoing research for vaccine development, existing current research offer only limited efficacy. Antiviral treatments, such as valacyclovir, exhibit promising results in reducing vertical transmission rates and improving outcomes in symptomatic neonates [9,10]. Given the potentially serious consequences of cCMV infection (non-hereditary hearing loss, neurodevelopmental disorders, and other permanent disabilities), early diagnosis and, above all, effective prevention are of paramount importance [11].

This review provides a comprehensive overview of cCMV infection, focusing on its epidemiology, pathogenesis, transmission, diagnosis, clinical manifestations, and preventive strategies, with the aim of highlighting new perspectives emerging from recent advances in CMV vaccine development.

## 2. Epidemiology of cCMV Infection

On a global scale, the incidence of cCMV infection is estimated to 0.6–0.7% of newborns, which equals approximately six to seven cases per 1000 births [3]. An analysis from countries with universal newborn screening programs indicates an overall prevalence of ~0.67%, with significant variation depending on geographic region [12]. The prevalence in high-income countries is ~0.3–0.5% (about one in two hundred newborns), whereas in low- and middle-income countries it is three times higher, around 1.4% [12,13]. This variation is attributed to higher seroprevalence rates (previous exposure to the virus and presence of IgG antibodies) in multigravidas, advanced maternal age, daycare workers, lower socio-economic status, and in racial and ethnic minorities [4,13].

The highest prevalence rates are recorded in regions of Sub-Saharan Africa; studies report cCMV prevalence ranging from ~1.4% (Ivory Coast) to 5–6% in some countries (e.g., Burkina Faso), which is well above the global average. In contrast, in countries such as the United States and Canada, the rate is ~0.5–0.8%, corresponding to approximately 30,000 newborns with cCMV annually in the U.S. [7].

Seroprevalence among women of reproductive age varies internationally from ~40–>90% (depending on hygiene conditions and socio-economic status) and affects the patterns of congenital infection [14]. In populations with low seroprevalence (e.g., developed countries), the majority of congenital infections is due to primary maternal infection during pregnancy. Conversely, in populations where nearly all women are already seropositive before pregnancy (e.g., many African and Asian countries), the majority (up to 75–90%) of cCMV cases arise from secondary infections (reactivation of the virus or reinfection of the mother) [13].

It is worth mentioning that previous maternal immunity to CMV does not eliminate the risk of vertical transmission; studies showed that congenital infection can still occur in immunized pregnant women, challenging the common belief that the risk was limited only to mothers with primary infection during pregnancy [13].

Primary maternal CMV is transmitted in utero to the fetus, with an average probability of ~30–40% [14,15]. The likelihood of CMV transmission increases with advancing gestational age at the time of maternal infection—approximately 30% in the first trimester, 45% in the second, and peaking at 72% in the third trimester [16]. On the contrary, in secondary infections, the risk of vertical transmission is much lower, around 1.2% [16]. Nonetheless, as preconception CMV infection is so common, approximately two-thirds of cCMV cases are attributed to either reinfection with a different CMV strain or reactivation of a latent infection [4]. In other words, prior immunity noticeably reduces but does not zero the possibility of congenital infection [7].

## 3. Diagnostic Evaluation

Congenital infection can be diagnosed prenatally or postnatally. Prenatally, the infection may be suspected after a documented MPI or, more commonly, after ultrasound findings suggestive of infection. Maternal CMV infection, primary or non-primary, is predominantly asymptomatic, and if otherwise, the symptoms and laboratory findings are non-specific. As a result, diagnosing CMV infection during pregnancy remains difficult, particularly given the lack of standardized routine prenatal screening [17,18,19,20].

### 3.1. Diagnosis of cCMV in Pregnancy

Serological testing can only diagnose MPI, and it is often unhelpful in non-primary infection (NPI). Routine antenatal serological screening is not recommended in most countries, and its application is limited to local or regional levels. In such settings, testing is offered only to pregnant women who present with signs indicative of CMV infection or findings in ultrasonography [8]. However, as women with primary infection in the first trimester are at the greatest risk for cCMV in their offspring and its sequelae (twenty-four-fold and six-fold higher than in the general population, respectively), these individuals could potentially benefit greatly from such a screening program [19]. According to the Consensus recommendations from the European Congenital Infection Initiative (ECCI), an initial serology is suggested as soon as possible, followed in seronegative women by a retest every 4 weeks until 14–16 weeks [20]. This is also indicated in all women with symptoms compatible with CMV infection and in the cases of abnormal fetal ultrasounds [21,22].

#### 3.1.1. Maternal Primary Infection—MPI

Serology screening in pregnancy is based on IgG and IgM testing followed by IgG avidity testing in cases of positive IgM. Serum samples collected 3–4 weeks apart, and tested in parallel for anti-CMV IgG, are pathognomonic for the diagnosis of primary infection. Seroconversion from negative to positive or to a significant increase (greater than fourfold [e.g., from 1:4 to 1:16]) in anti-CMV IgG titers is indicative of infection [18]. In addition, the use of IgG avidity assays, which measure the affinity maturation of the IgG antibody, combined with IgM titers, allow for improved identification of primary infection (sensitivity of 92%) when compared with standard serial serologic assays [18,23]. Therefore, the identification of high-avidity IgG in the first trimester provides strong evidence against a primary CMV infection occurring during early gestation, the periconceptional phase (around two weeks before or after conception), or the preconceptional window (2–8 weeks before conception). This distinction is clinically significant, as the risk of vertical transmission during these intervals is estimated at 36.8%, 21%, and 5.5%, respectively [20]. The ECCI [24] recommend that sera with an intermediate avidity result be retested with another avidity assay or be sent to a reference laboratory. A high IgG avidity index on the second assay is strongly suggestive of a past infection and effectively rules out recent primary maternal CMV infection. Low or confirmed intermediate IgG avidity in the first trimester, in combination with positive IgM, is suggestive of MPI [25,26]. It should be noted that the presence of CMV IgM alone is inadequate finding for the diagnosis of primary infection as there is a 90% false-positive rate due to the lack of specificity of IgM antibodies, their presence in the serum for extended period (up to 6–9 months and occasionally exceeding 12 months), the possible cross-reactivity with other viral infections, their presence in non-primary infection, and the fact that they can be a result of nonspecific polyclonal stimulation of the immune system [18,19,27]. CMV polymerase chain reaction (PCR) in whole blood is highly sensitive (up to 100%) for diagnosing recent MPI within the first 2–4 weeks. However, its specificity is low for timing, as DNAemia can persist for up to a year. Urine PCR also declines over time and is unreliable for the determination of the time of infection. CMV PCR testing in whole blood demonstrates high sensitivity—approaching 100%—for the detection of recent MPI, particularly within the first 2–4 weeks post-infection [19,20].

#### 3.1.2. Maternal Non-Primary Infection—MNPI

At present, no reliable laboratory test exists to determine which women with preexisting CMV immunity are at risk of transmitting the infection to their fetus. Both serological methods and DNA detection by PCR in blood, urine, or saliva are used, but with low reliability. Therefore, the only way of confirming non-primary CMV infection (whether reinfection or reactivation) is by invasive testing [19].

### 3.2. Diagnosis of Fetal Infection

After confirmation of maternal infection or suspicion of fetal infection, the virus can be identified in the amniotic fluid of the index pregnancy, using either viral culture or PCR techniques [28]. Fetal blood sampling is not recommended due to its lower sensitivity and potential risks for the fetus. PCR remains the gold standard method for diagnosis of fetal CMV infection, with the highest sensitivity and specificity. However, a recent meta-analysis reported false-negative rates as high as 8%, which may be due to delayed vertical transmission resulting in fetal infection occurring later in pregnancy, often without significant clinical consequences [29]. To improve diagnostic accuracy in cases of maternal infection, it is advised to delay amniocentesis by 6 to 8 weeks after maternal seroconversion, which is the peak of maternal viremia followed by placental infection and ultimately fetal dissemination [29,30], or until after 17–21 weeks of gestation, the developmental stage when fetal renal function is established and the virus is excreted into the amniotic fluid [31]. PCR from chorionic villus samples could help diagnose early MPI after 12 weeks, and exclude CMV-related embryopathy, though long-term data are needed to confirm this [19,32]. Although the results of these techniques are extremely valuable for the diagnosis of congenital infection, they offer limited insight into the assessment of disease severity [18].

### 3.3. Antenatal Ultrasound Findings

In the absence of routine universal serological screening, prenatal ultrasound serves as the primary tool for raising suspicion for fetal infection, and prompts further investigation [18,33]. Prenatal ultrasound may reveal a wide range of abnormalities in fetuses infected in utero by CMV. One of the most consistent sonographic findings is bilateral periventricular hyperechogenicities, which are typically attributed to periventricular calcifications and are considered highly suggestive of CMV-related central nervous system involvement [34,35]. Placental abnormalities (2% of cases) include placentomegaly and heterogeneous placental texture, which may reflect underlying inflammation or infarction [8,33,36]. Hamilton et al. [37] demonstrated that CMV infection alters the immunologic milieu of the placenta, likely through the induction of pro-inflammatory cytokines, such as monocyte chemoattractant protein-1 (MCP-1) and tumor necrosis factor-alpha (TNF-α), which contribute to placental injury.

A wide range of ultrasonographic findings may be associated with fetal cCMV infection, although most are non-specific. Cranial and central nervous system (CNS) anomalies are among the most characteristic, including ventriculomegaly (6.1%), intracranial and periventricular calcifications (6.3%), a hyperechogenic periventricular halo (3%), cerebellar hypoplasia, enlargement of the cisterna magna, and neuronal migration disorders such as polymicrogyria, pachygyria, and lissencephaly (<1%) [8]. Microcephaly (6%) and hydrocephalus (3.6%) are also reported and are often correlated with more severe neurodevelopmental outcomes [8]. Abdominal findings may include hepatosplenomegaly (3.8%), echogenic bowel (13%), hepatic calcifications (1.2%), and other intra-abdominal calcifications. In some cases, abnormal fluid accumulation such as ascites (4.2%), pleural effusion (1%), pericardial effusion (1%), and intra-abdominal pseudocysts are observed [8]. Hydrops fetalis occurs in approximately 1% of cases, and oligohydramnios (3.4%) and polyhydramnios (1%) may be present rarely. Additionally, fetal growth restriction (IUGR) or a small for gestational age (SGA) neonate occurs in about 9% of affected fetuses [8].

If present isolates are non-specific, a combination of multiple abnormalities, particularly CNS calcifications and evidence of growth restriction, should raise strong suspicion for cCMV infection. This is especially pertinent in the presence of supportive maternal serology or positive amniotic fluid PCR result [3,33,38,39].

### 3.4. Prenatal Predictors of cCMV Outcomes

Following a confirmed or highly probable CMV infection, the prognostic assessment of prenatal findings is of paramount importance for the subsequent management of pregnancy [28,40]. The timing of maternal infection is a critical determinant, particularly when evaluated with additional parameters such as serial ultrasound assessments during the second and third trimesters, fetal platelet count obtained via chordocentesis, and prenatal magnetic resonance imaging (MRI), all of which serve as important predictive markers for identifying affected fetuses [41].

According to current evidence, specific prenatal findings during the second and third trimesters have substantial prognostic value in fetuses with cCMV infection [20,33,42]. A normal ultrasound scan after the second trimester is associated with a negative predictive value exceeding 90% for adverse neonatal outcomes [43,44]. In contrast, the presence of severe cerebral abnormalities on ultrasound is strongly associated with poor prognosis. Extracerebral abnormalities confer an intermediate risk, with studies indicating a 20–60% probability of long-term sequelae [45,46]. Moreover, significant hematological disturbances, particularly fetal thrombocytopenia, were linked to unfavorable outcomes [43,47,48,49]. When these findings co-occur from the second trimester onward, the risk of either symptomatic congenital infection or termination of pregnancy due to severe cerebral injury rises to approximately 60% [43]. MRI at 32 weeks of gestation with serial ultrasound assessment enhances the prognostic evaluation of fetal CMV infection during the first trimester [41]. This combined diagnostic approach demonstrates a high negative predictive value, nearing 100%, for identifying symptomatic cases at birth and predicting the development of moderate to severe sequelae. However, there remains a residual 17% risk of SNHL [50].

### 3.5. Diagnosis in the Neonate

Indications for cCMV testing include clinical signs suggestive of MPI, suspicious findings on prenatal ultrasonography or fetal MRI, as well as clinical manifestations in the neonate, especially SNHL [20]. At the moment, routine screening of newborns for CMV infection is not currently recommended; however, some states have adopted targeted testing for infants who fail the newborn hearing screening [51,52]. According to the recent guidelines, symmetric IUGR should prompt consideration for screening for cCMV, along with other high-risk groups such as very preterm neonates (<32 weeks of gestation) and infants with very low birth weight (<1500 g), especially when there is uncertainty regarding the timing of infection (i.e., congenital vs. postnatal) [20]. After birth, the diagnosis is confirmed by PCR testing of urine or saliva within 3 weeks. Early sample collection is critical, as delayed testing may hinder the distinction between congenital and postnatally acquired CMV infection [1]. A positive CMV PCR results on a saliva sample should be confirmed with a CMV PCR on a urine sample, since saliva can be contaminated from the genital tract or the last breastfeeding and as a result lead to a false positive test [20]. Detection of CMV DNA on the neonatal dried blood spot (DBS) is considered the gold standard for retrospective diagnosis of cCMV, but it may miss some cases (84.4% sensitivity and 100% specifity) [53]. PCR testing on DBS offers a practical approach to cCMV screening, since samples are routinely collected in newborn programs. However, according to the latest studies, its sensitivity has historically been low (≈28–42%) compared with saliva or urine PCR, leading to under-diagnosis [54,55]. Recent assay improvements, including droplet digital PCR and direct PCR, have increased detection rates to 73–77%, with combined data showing up to 85% sensitivity [56]. Detection is reliable at high viral loads, but inconsistent at low copy numbers, which is particularly relevant for asymptomatic infants. A large real-world study from Minnesota, the first state to mandate universal screening, confirmed saliva PCR’s high sensitivity (93.4%) and DBS PCR’s moderate but practical performance (72.4%), with several infants later developing delayed-onset hearing loss [57]. Quantitative PCR can measure CMV viral load, yet studies show no clear correlation between DBS viral levels and outcomes such as sensorineural hearing loss [55]. Thus, while DBS PCR is feasible and informative, its limited sensitivity and prognostic value warrant cautious use in universal screening. CMV serology is not recommended in neonates for screening or diagnostic purposes, as only a negative CMV IgG result in either the mother or the newborn reliably excludes congenital infection [20,58,59].

## 4. Pathogenesis

HCMV is a complex virus with a large genome, the biggest among all human herpesviruses, and it exhibits strict host specificity [60]. Human CMV infections stem from the virus-mediated evasion of T-cell immune responses [16]. The virus lyses host cells, spreads to different cells and tissues due to its broad cell tropism [61] and remains dormant within the host. Key aspects of HCMV underlying its diverse clinical associations are viraemia, the threshold-dependent link between viral burden and disease, and immune pressure that maintains viral persistence within sanctuary sites [62].

The virus disperses throughout the human body by infecting the white blood cells and vascular endothelial cells. Research on HCMV replication rates has shown that its kinetics are brisk, with a doubling period of viraemia in the region of one day [63]. This rapid replication is comparable to primary human immunodeficiency virus (HIV) infection, indicating that the common perception of HCMV as a slow-growing virus stems from early studies in fibroblast cultures, which do not accurately reflect in vivo infection dynamics [63,64]. In fibroblasts, the virus replication cycle generally lasts about 72 to 96 h [65]. The virus interferes with the recognition and processing of viral antigens by major histocompatibility complex (MHC) class I molecules, induces abnormal helper T-cell responses by degrading MHC class II molecules, and inhibits the activity of host natural killer (NK) cells [16] hereby evading the immune response. The duration and efficacy of HCMV proliferation can differ relying on factors like cell type, viral strain, and multiplicity of infection. Notably, the virus is capable of establishing latency in specific cell types, most prominently in CD34+ hematopoietic progenitor cells and CD14+ monocytes [65]. Throughout the latent period, HCMV shows highly limited gene expression, with only a small set of transcripts being produced.

The immune system devotes more assets to regulating HCMV than any other virus, as healthy seropositive hosts usually dispose over 1% of their peripheral blood T cells targeting a single HCMV antigen [62]. This creates a ‘stand-off’ scenario: the virus remains controlled under normal immune conditions but can rapidly expand if the immune response is impaired, as occurs in immunosuppressed patients, HIV-infected individuals, neonates, or critically ill patients [64]. Thus, once the settled immune system is compromised, the opportunist virus is ready to replicate rapidly, leading to productive infection and potential clinical manifestations. Indicative situations are patients receiving immunosuppressive therapy to restrain graft rejection, HIV positive people, or individuals with immature immune systems, such as developing fetuses and bone marrow transplant recipients with newly engrafted marrow. It may also occur if the immune system is abruptly overwhelmed by a severe physiological shock leading to intensive care admission. The virus’ ability to persist in sanctuary sites under immune surveillance highlights the importance of both viral replication dynamics and host immune competence in determining the outcome of HCMV infection [64].

During pregnancy, CMV is transmitted hematogenously from the mother to the developing fetus through the placenta [66]. It is transmitted through the syncytiotrophoblast layer that surrounds the chorionic villi, leading to disrupted placental function and impaired fetal development. Moreover, infection of trophoblast cells by CMV disrupts the normal process of interstitial implantation, providing a plausible explanation for the increased risk of early pregnancy loss observed in affected pregnancies [16].

In addition to transplacental transmission, neonates can also acquire the HCMV virus perinatally through direct contact with infected epithelial cells or genital secretions during vaginal delivery [67], or postnatally via maternal milk. CMV is detected in breast milk (especially colostrum) in a seropositive mother and can be transmitted to the breastfeeding offspring [68]. Symptomatic HCMV infection caused by breast milk is uncommon in full-term infants, due to the transfer of protective maternal antibodies beginning in the third trimester, around the 29th gestational week [69,70]. However, infants born very preterm lack this antibody shield, and that renders them highly vulnerable to symptomatic HCMV infection via breast milk. Additional risk factors for developing symptomatic or severe infection include low birth weight and early postnatal exposure [70,71,72].

The most common way of acquiring CMV is community transmission via persistent and prolonged viral shedding by asymptomatic individuals, especially toddlers, through infected body fluids, including saliva, urine, tears, etc. This is a major route for women of reproductive age, including parents and daycare workers [7]. CMV can also be acquired by sexual intercourse (mainly unprotected) via genital (semen, vaginal) secretions [24]. It is considered a sexually transmitted infection, especially among young adults.

Transmission can also occur via solid organs or hematopoietic stem cell transplantation from seropositive donors [73]. The risk of CMV reactivation or primary infection post-transplant is high, especially without prophylactic measures. Rarely, CMV can be spread via blood transfusion through infected leukocytes, with the higher risk among immunocompromised individuals (e.g., transplant recipients, HIV patients, as well as very preterm neonates) [74,75,76].

### 4.1. CMV Infection During Pregnancy

During pregnancy, a woman may experience a primary CMV infection, a reinfection with a different strain of CMV, despite natural immunity, or a reactivation of the latent virus, during which viral replication is triggered, usually due to stress or other medical conditions [4]. These possible cases differ significantly in terms of the mother’s immune response and the consequences for the fetus.

### 4.2. Immune Response

Upon HCMV infection, the innate immune system provides the first line of defense. Myeloid cells, including monocytes, macrophages, and dendritic cells (DCs), sense the virus via pattern recognition receptors (PRRs) such as toll-like receptors (TLRs) and produce cytokines that shape adaptive responses [77]. HCMV evades myeloid cell responses by downregulating MHC class I/II and costimulatory molecules, yet limited infection of DCs in vivo allows T-cell activation [78]. It is well known that fetal myeloid cells produce lower amounts of proinflammatory cytokines and favor Th2 responses, which may protect against immunopathology but reduce viral control [79,80,81].

Fetal and neonatal NK cells, which play a key role in controlling HCMV, are immature, hyporesponsive, and influenced by TGF-β, limiting cytotoxicity [82,83]. Expansion of “adaptive” NKG2C+ NK cells occurs in seropositive children, particularly after symptomatic infection, reflecting interaction with T-cell responses [84,85]. Decidual NK cells in pregnancy contribute to placentation and may acquire cytotoxic potential upon encountering HCMV-infected cells [86,87]. The CD8+ T cells provide cytotoxic activity, whereas CD4+ T cells mediate helper and regulatory functions [88]. Neonatal T cells show limited clonal diversity, reduced multifunctionality, and Th2-biased responses, affecting viral clearance [89,90,91]. Congenital HCMV induces early CD8+ T-cell responses by 22 weeks gestation; γδ T cells expand in utero but their role remains unclear [92,93,94].

Neonates have predominantly naïve B cells with limited affinity maturation [95,96]. Maternal HCMV-specific IgG antibodies cross the placenta, partially protecting the fetus, though pre-existing immunity does not fully prevent transmission or reinfection with new strains [97,98,99,100]. Antibodies targeting the pentameric complex are particularly protective [101,102]. Passive immunization with hyperimmune globulin or maternal IgG reduces viral load in experimental models [103,104].

In primary infection, the pregnant woman is seronegative, and viral replication is faster before the immune response is triggered. The pregnant woman produces IgM antibodies and subsequently generates specific IgG antibodies, initially of low avidity, which increase in titer over the course of several weeks. In contrast, in a secondary infection, the pregnant woman already carries circulating high-avidity IgG antibodies and memory cellular immunity against the virus. This means that viremia is usually resolved more quickly, while maternal infection is often asymptomatic or subclinical. The presence of maternal IgG antibodies that cross the placenta may also partially protect the fetus by reducing the severity of fetal infection, in the case it occurs [15]. Nevertheless, pre-existing immunity is only partially protective—it does not completely prevent transmission nor guarantees the absence of harmful effect [7]. In recurrent infection with a different strain, the virus can partially trigger existing immunity, and a new immune response occurs. In the laboratory, differentiation between primary and secondary infection is based on the detection of IgM and IgG avidity testing: low-avidity IgG indicates a recent primary infection, while high-avidity IgG alongside IgM antibodies can be indicative of either recent reinfection or reactivation of latent CMV [105].

## 5. Clinical Status

Most neonates with cCMV are asymptomatic at birth and may never develop clinical manifestation and health complications [68]. Nevertheless, some of these infants can experience significant sequelae, either early in life or later, potentially resulting in long-term disabilities. Approximately one in two-hundred babies are born with cCMV and out of them, one in fice babies will have birth defects or long-term health problems, like hearing loss [61]. CMV can also be suspected prenatally from ultrasound findings in the developing fetus [18]. The fetus may acquire CMV infection asymptomatically or present with clinical signs in utero. In the most severe cases, cCMV can result in pregnancy loss, or neonatal death occurring in approximately 3–4% of symptomatic newborns [1,8].

Congenital infection resulting from a primary maternal infection, especially if it occurs early in pregnancy, has a higher likelihood of leading to more severe sequelae in the offspring. It is estimated that ~10–15% of fetuses infected by primary maternal infection exhibit symptoms at birth, and many of these will experience long-term disabilities [7,16]. In non-primary infections, the majority of congenitally infected fetuses are asymptomatic at birth and may later develop issues, primarily SNHL that manifests in early childhood [7].

Secondary infections used to be considered negligible, but recent data suggest that prior maternal immunity does not entirely shield the newborn from developing adverse neurological conditions [66]. In a study comparing symptomatic newborns, those born to mothers with secondary infection showed similar rates of neurological complications and unilateral hearing loss compared to those born to mothers with primary infection (48.1% vs. 33.9%, respectively) [106]. These results are similar to a preceding study which reported 46.2% symptomatic newborns to mothers with primary infection and 60% symptomatic newborns to mothers with secondary infection [107]. However, more severe manifestations such as bilateral deafness and extensive brain damage occur mainly in primary infection [106]. The most significant risk factor for symptomatic cCMV at birth and lasting complications is the transmission of a primary maternal infection to the fetus earlier in pregnancy [108]. The clinical spectrum of the disease in the neonate, including immediate manifestations and long-term sequelae, appears similar following both primary and non-primary maternal CMV infections [106,107]. This observation suggests that pre-existing maternal immunity offers only partial protection against fetal infection and its consequences.

### 5.1. Clinical Manifestation Depending on the Timing of Maternal Infection

#### 5.1.1. First Trimester Maternal CMV Infection

The risk of severe cCMV infection increases in early pregnancy [3,18,20]. While the probability of transmission increases with gestational age, serious neonatal consequences are essentially limited to first-trimester infections. A recent meta-analysis [109] reports that the percentage of fetal impairment progressively decreased, from 28.8% at the periconception period to 19.3% in the first trimester, 0.9% in the second trimester, and 0.4% at the third trimester. From the fetuses that were infected, symptoms occurred in 1.3% for maternal infection in the periconception period, 9.1% in the first trimester, 0.3% in the second trimester, and 0.4% in the third trimester [109].

Newborns that were affected at the embryonic stage may present microcephaly and CNS damage with severe neurodevelopmental sequalae. This is due to CMV-induced disruption of neural precursor cell proliferation, differentiation, and survival, which occurs at a critical window of brain development (from 4 weeks gestation onward) [109]. Specifically, the meta-analysis by Chatzakis et al. [109], demonstrated that central nervous system (CNS) abnormalities detected by ultrasound occurred in 10.0% of cases following maternal infection in the first trimester, compared to 0.3% and 0.5% after infections in the second and third trimesters, respectively. Notably, brain lesions occur only with first-trimester infections and are not seen after later exposures [32,41], while microcephaly may correspond to infection at <18 weeks’ gestation [44,109].

SNHL is a major complication associated with early cCMV infection. Maternal infection during the first trimester poses the highest risk, often resulting in bilateral and severe hearing loss in the infant [54]. In one cohort, 32% of infants infected in the first trimester developed SNHL, and/or neurologic deficits by the age of 2 years [41]. Likewise, Chatzakis et al. [109] demonstrated that the risk of severe SNHL or neurodevelopmental impairment declines significantly with advancing gestational age at the time of maternal infection, from 22.8% in the first trimester to 0.1% in the second trimester and 0% in the third trimester. It is worth noting that milder forms of SNHL were observed even when infection occurred after the first trimester; however, cases of profound hearing loss or complete deafness seem to be confined primarily to infections acquired during the first trimester [110,111].

About half of symptomatic cCMV infants have growth restriction [30]. First-trimester CMV infection may disrupt early placental and fetal development, contributing to outcomes such as low birth weight and small-for-gestational-age status [112]. It has also been associated with preterm birth and stillbirth, likely through its impact on placental function and development. However, the exact mechanisms by which CMV induces these adverse outcomes remain incompletely understood. Several mechanisms have been proposed based on in vitro studies employing laboratory-adapted viral strains and diverse human cell models. These effects include reduced invasiveness of extravillous trophoblasts, disruption of Wnt signaling pathways in cytotrophoblasts, tumor necrosis factor-α (TNF-α)-induced apoptosis of trophoblast cells, CMV-mediated changes in placental cytokine profiles, inhibition of indoleamine 2,3-dioxygenase (IDO) activity, and downregulation of class I MHC molecules on trophoblasts [112]. A major limitation in this field remains the lack of appropriate in vivo animal models that faithfully replicate human placentation and CMV pathophysiology. Nevertheless, current evidence provides valuable insights into the potential mechanisms by which CMV disrupts placental development and function, contributing to adverse pregnancy and birth outcomes [112].

Roughly 60% of symptomatic infants present with hepatosplenomegaly, often accompanied by jaundice, and ~75% have petechiae or “blueberry muffin” rash due to thrombocytopenia [30]. Other signs can include pneumonitis, anemia, and neonatal hydrops in severe cases [113,114]. The earlier the fetal brain is exposed to CMV, the more severe the clinical manifestations at birth, particularly in terms of neurodevelopmental impairment [115].

#### 5.1.2. Second and Third Trimester Maternal Infection

While first-trimester infection carries a high risk of sequelae, in later gestation (second to third trimester)—although the transmission rates are higher—clinical symptoms at birth are milder or absent in most cases [3,115,116]. CNS development is more advanced and profound, thus structural disruption is infrequent [19]. Still, subtle neurodevelopmental or auditory deficits can be evident postnatally [29,109]. Miller et al. [116] reported that about 7% of children with second-trimester cCMV had a mild adverse outcome a few years later, typically partial unilateral hearing loss or slight motor/language delays, and none had severe deficits. No cases of extensive neurologic damage or bilateral deafness were observed in that group. Τhird-trimester cCMV infection rarely produces any symptomatic disease at birth or profound early consequences [108,109]. Only a single case of transient mild motor delay among 28 infants infected in late third trimester was reported [116]. Consequently, third trimester infection is associated with the most favorable prognosis in the neonate.

### 5.2. Signs That Can Appear Soon After Delivery

cCMV infection may affect various systems. Signs that can be present soon after birth include a petechial or purpuric rash observed in approximately 50–70% of affected neonates, and cholestatic jaundice in 40–70% of cases [117,118,119]. Neurological manifestations may include microcephaly (35–50%), SNHL (present at birth in approximately 35%), hypotonia and/or lethargy (30%), seizures (5–10%), and poor feeding, or diminished suck reflex (20%) [120]. In some infants, the initial presentation is primarily characterized by CNS findings (primary neurophenotype) with absence of the typical somatic signs and phenotypically healthy at birth or with mild microcephaly. In such cases a strong clinical suspicion is needed to early identify cCMV infection, which is particularly important given that these presentations may progress to significant neurologic impairment (e.g., global developmental delay, abnormal muscle tone, hemiparesis, seizures) and microcephaly [121]. Cases resembling inherited leukodystrophies were described, and when neuroimaging is undertaken, findings often include polymicrogyria or other types of cortical dysplasia [122]. Growth abnormalities such as IUGR or small for gestational age (SGA) status are noted in 40–50% of neonates [113]. Ocular manifestation can involve chorioretinitis (10–15%), a sign related with adverse long-term neurodevelopmental outcomes, retinal scarring, optic nerve atrophy, central visual impairment, and strabismus [123,124]. Additional systemic findings may encompass hepatosplenomegaly (40–60%), and pneumonia (5–10%). Cardiac involvement is uncommon in symptomatic newborns. Although endocrinopathies and renal abnormalities have been reported, their direct association with cCMV infection remains uncertain and requires further investigation [125,126,127].

Preterm birth is also commonly associated with symptomatic congenital infection observed in 25–35% of cases. Very preterm neonates (<32 weeks of gestation) more commonly present with pneumonitis, signs of viral sepsis, thrombocytopenia, and coinfections and less commonly have microcephaly or intracranial calcifications compared with term neonates [3,19,113]. Life-threatening manifestations appear in 8–10% of symptomatic neonates and may include sepsis-like illness, myocarditis, viral-induced hemophagocytic lymphohistiocytosis, and/or other severe end-organ involvement [113]. Premature infants and infants with primary T- or natural killer-cells immune disorders are at greatest risk for mortality from cCMV. The mortality rate of these infants can be up to 30%; in contrast, the overall mortality rate among infants with cCMV ranges from 4 to 8% during the first year of life [113].

The clinical presentation of neonates was further classified depending on the severity of the symptoms [105,128]. In both frameworks, severe disease is defined primarily by CNS involvement [3,126]. A European Expert Consensus Statement on the diagnosis and management of cCMV recommends stratification based on symptom severity, particularly in cases presenting with abnormal neurological or ophthalmologic findings, microcephaly, or neuroimaging abnormalities. These imaging features may include intracranial calcifications, moderate to severe ventriculomegaly, periventricular cysts, white matter abnormalities, cerebral or cerebellar hypoplasia, hippocampal dysplasia, and neuronal migration disorders [128]. They also consider severe single-organ disease, such as liver failure or hepatosplenomegaly, as well as multiorgan involvement. Infants with transient or clinically insignificant findings that resolve spontaneously within weeks are excluded from this group [128]. Rawlinson et al. [105] similarly define severe disease by the presence of CNS involvement, including microcephaly, ventriculomegaly, calcifications, periventricular echogenicity, cortical or cerebellar malformations, and detection of CMV DNA in cerebrospinal fluid, as well as systemic manifestations such as thrombocytopenia, petechiae, hepatosplenomegaly, IUGR, or hepatitis.

According to European Expert Consensus Statement on the diagnosis and management of cCMV, moderate disease in the Luck et al. classification encompasses a heterogeneous group of findings, including persistent hematologic or biochemical abnormalities lasting more than two weeks, or the coexistence of multiple mild disease features [128]. Rawlinson et al. [105], by contrast, do not separate moderate and severe presentations, instead they group them together as a single category. In terms of mild disease, European Expert Consensus Statement on the diagnosis and management of cCMV [128] describe cases with one or two transient and clinically insignificant abnormalities such as petechiae, mild hepatosplenomegaly, or mild deviations in laboratory values (e.g., thrombocytopenia, anemia, leukopenia, borderline liver enzymes, or conjugated hyperbilirubinemia), as well as SGA neonates without microcephaly. Rawlinson et al. [105] similarly characterize it as a mild disease when it involves one or two isolated manifestations that are mild and transient, including mild hepatomegaly or a single elevated laboratory parameter. The isolated SNHL category is described only by Rawlinson et al. [105], defined as SNHL with a threshold of ≥21 dB without other accompanying abnormalities. Finally, asymptomatic cases are defined as these with no evident clinical signs or symptoms at birth [3,128], and according to the Rawlinson classification [105], normal hearing is a specific criterion for this category.

#### 5.2.1. Associated Laboratory Abnormalities

Associated laboratory abnormalities in cCMV infection include elevated liver transaminases (50–83%), thrombocytopenia (48–77%), and direct and indirect hyperbilirubinemia (36–69%) [113]. Hematologic abnormalities, such as hemolytic anemia (reported in 5–10% of cases), neutropenia, lymphopenia, lymphocytosis, thrombocytosis, and leukemoid reaction are less frequently encountered. In neonates undergoing lumbar puncture, cerebrospinal fluid (CSF) analysis may reveal elevated protein concentrations (46% in one case series by Boppana et al. [118]) indicative of CNS involvement [3,68,113,119].

#### 5.2.2. Neuroimaging Abnormalities

Neuroimaging findings in cCMV infection are diverse and may involve multiple structural abnormalities. Commonly reported anomalies include ventriculomegaly (10–53%), periventricular calcifications (34–70%), lenticulostriate vasculopathy (27–68%), and white matter abnormalities seen in 22–57% of cases [113,129,130,131]. Additional findings may comprise periventricular leukomalacia and cystic changes, particularly germinolytic cysts or anterior temporal lobe cysts in 11% of cases, as well as ventricular septations, adhesions, cerebral atrophy, dysgenesis of the corpus callosum, and cerebellar hypoplasia. In more severe cases, imaging may reveal features consistent with disrupted neuronal migration, such as lissencephaly, polymicrogyria, and cerebellar malformations (10–38%) [130,131,132]. Neuroimaging abnormalities detected by brain CT and/or MRI are critical in guiding treatment decisions and in assessing long-term neurodevelopmental prognosis in infants with cCMV infection [133]. The most severe manifestations of CNS injury are typically associated with first-trimester acquisition of CMV by the developing fetus [3,113].

### 5.3. Long-Term Complications

Approximately 17–19% of individuals with cCMV infection develop long-term sequelae. This proportion increases substantially in cases of MPI during the first trimester, where 51–57% of affected neonates present with symptomatic disease and subsequent neurodevelopmental impairment [8]. The gestational age at the time of maternal infection is the most significant predictor of long-term sequelae in cCMV. Although the association between the time of infection and clinical outcome was unclear, advances in accurate determination of the time of MPI through serological methods clarified this relationship. Recent evidence indicates that long-term sequelae occur exclusively in infants infected following first-trimester MPI (before 14 weeks of gestation), whereas no persistent complications were reported in cases where the primary infection occurred during the second or third trimester [3]. Reported long-term sequelae of cCMV infection include intellectual impairment, global neurodevelopmental delay, motor dysfunction, epilepsy, and microcephaly—often reflecting underlying cortical malformations [3,113]. Epilepsy occurs in approximately 10% of infants with symptomatic cCMV infection and is frequently accompanied by other signs of CNS involvement, such as neuronal migration abnormalities and ventricular enlargement [134]. Children with symptomatic infection at birth have a 40–70% risk of neurological sequelae [3]. Additional neurodevelopmental complications may involve autism spectrum disorder, cerebral palsy, coordination deficits, and muscular weakness. Children with cCMV infection exhibited vestibular and balance impairments, regardless of whether they also present with hearing loss [135]. Sensory impairments are also common, particularly sensorineural hearing loss, the most frequent long-term complication, as well as a range of visual deficits including chorioretinitis, strabismus, cortical visual impairment, and optic atrophy. Ocular abnormalities, mainly chorioretinitis with retinal scarring, are seen almost exclusively in neonates symptomatic at birth, with reported prevalence ranging widely from 0 to 40% [3]. A recent study involving a small cohort of 34 patients reported that cCMV infection is linked to impaired olfactory function in children who were symptomatic at birth [29,136]. Dental anomalies were also described, likely reflecting intrauterine disruption of developing tissues. The overall reported mortality rate was 0.5%, indicating that fatal outcomes are relatively uncommon [3,113]. The diversity and severity of these outcomes highlight the critical need for early diagnosis, targeted neonatal monitoring, and multidisciplinary long-term care for children with cCMV infection.

Hearing loss is the most common complication and can appear at birth or develop later in life, even in babies that had passed the screening hearing test. For this reason, extra attention should be given to babies with cCMV, and regular hearing checks must be scheduled [68]. SNHL is a frequent long-term complication of cCMV infection, detected in a significant proportion (one-third to one-half) of infants exhibiting clinical signs of infection. While SNHL can be present at birth, it may present after the neonatal period in 18–30% of symptomatic cases [113]. According to a systematic review [137], bilateral hearing impairment was identified in 71% of children with symptomatic infection, in contrast to 43% among those with asymptomatic cCMV. Furthermore, hearing loss in symptomatic individuals often follows a progressive course, with deterioration documented in 18–63% of cases and ultimately advancing to severe or profound levels in 78% of affected ears [113].

### 5.4. Asymptomatic Infection

About 90 percent of congenitally infected infants will be asymptomatic at birth [138,139]. Newborns with asymptomatic cCMV infection may exhibit subtle clinical differences, such as mildly reduced birth weight and slightly earlier gestational age compared to uninfected infants [113]. Ocular involvement, especially retinal lesions and strabismus, is observed in 1–2% of cases, although such findings are infrequently associated with significant visual impairment. Furthermore, neuroimaging abnormalities including periventricular leukomalacia, ventriculomegaly, and punctate intracranial calcifications were documented in 5–20% of neonates with otherwise asymptomatic cCMV infection. Despite the absence of overt clinical signs at birth, SNHL develops in approximately 10–15% of these infants [113].

Isolated SNHL in the absence of any other clinical, laboratory, or neuroimaging findings, is considered by some as asymptomatic cCMV and by others as a manifestation of symptomatic infection [3]. An international consensus committee as well as the Red Book Committee of the American Academy of Pediatrics [140] agree that isolated SNHL is part of the asymptomatic infection, whereas the European Society for Paediatric Infectious Diseases expert consensus panel [20] states that isolated CMV-associated SNHL is included in the clinical manifestations of cCMV infection involving the CNS [54].

## 6. Therapeutic Approaches and Clinical Management of Fetuses and Neonates Diagnosed with cCMV

**Figure 1:**
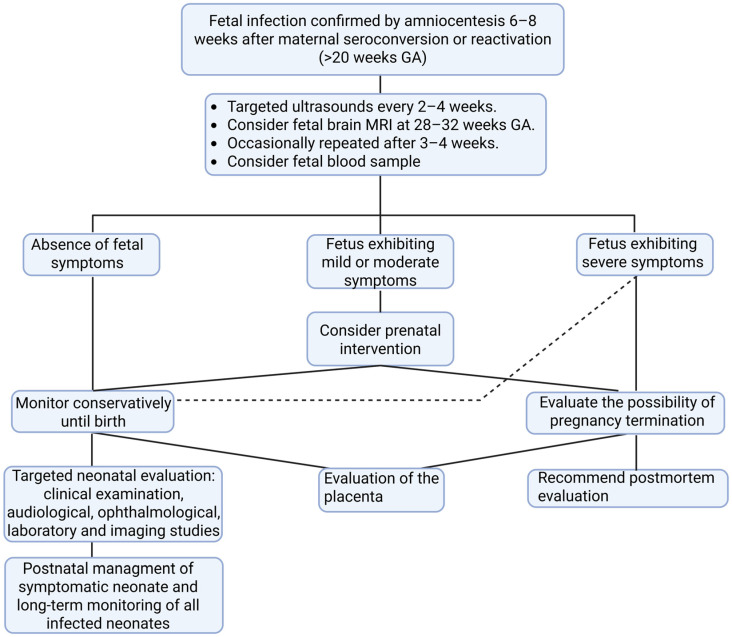
Approach for managing cCMV infection. (Created in BioRender. L., A. (2025) https://BioRender.com/3hd6qkj (accessed on 24 July 2025).

A pregnant woman with confirmed fetal CMV infection—typically diagnosed via amniocentesis—requires close and targeted prenatal monitoring. Serial ultrasound examinations are performed throughout the remaining pregnancy to assess evolving signs of fetal compromise, including growth restriction, ventriculomegaly, ascites, or other markers of CMV-related pathology [18,140]. Figure 1 presents a structured follow-up guide for managing pregnancies complicated by confirmed cCMV infection, aiming at supporting individualized decision-making and optimizing perinatal outcomes.

**Figure 2:**
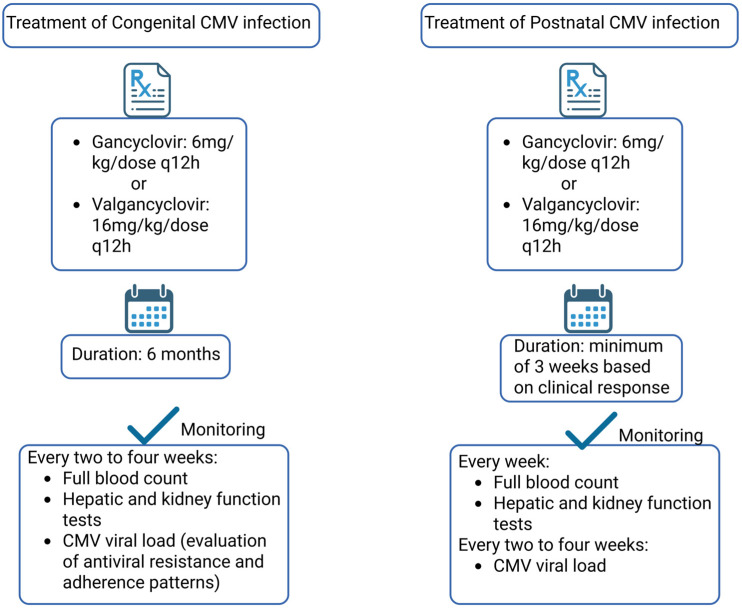
Therapeutic interventions for CMV infection. (Created in BioRender. L., A. (2025) https://BioRender.com/cxnwlug (accessed on 24 July 2025).

Newborns diagnosed with coCMV infection undergo a comprehensive evaluation to determine the severity of disease—categorized as asymptomatic, mildly, moderately, or severely symptomatic [141]. This assessment is critical, as it guides prognosis and therapeutic decisions. It typically includes a detailed physical examination, audiologic testing (audiogram), neuroimaging (cranial ultrasound, MRI, or CT), baseline laboratory investigations (including complete blood count and liver function tests), and, when indicated, an ophthalmologic examination to detect chorioretinitis or other vision-threatening abnormalities. In neonates with symptomatic cCMV infection, a six-month course of valganciclovir is currently the recommended treatment [20]. While short-term adverse effects such as neutropenia and hepatotoxicity are reversible, they necessitate close monitoring through frequent laboratory evaluations [142]. More concerning, though based on animal studies, are the potential risks of long-term gonadal toxicity and carcinogenicity, for which human long-term safety data are still not available [137]. Randomized controlled trials demonstrated that valganciclovir effectively reduces the risk of hearing loss and neurodevelopmental impairment, with a six-month treatment regimen showing superior outcomes compared to a previously established six-week course [143,144]. At present, antiviral therapy is not recommended for asymptomatic neonates with cCMV infection [145]. Figure 2 illustrates the recommended duration and dosing regimen of antiviral therapy for newborns with cCMV, with treatment protocols differing based on whether the infection is congenital or acquired postnatally. These tailored therapeutic strategies reflect the distinct clinical courses and risks associated with each type of infection. Optimizing treatment duration and dosage is critical to maximize efficacy and minimize potential toxicity, underscoring the importance of individualized management plans in neonatal CMV infection.

## 7. Prevention of cCMV Infection According to the Latest Guidelines

In the general population CMV infection is typically asymptomatic, but congenital infection can result in permanent consequences for the newborn. For this reason, preventing cCMV infection is crucial and this has created significant research interest. At present, prevention relies primarily on behavioral, educational and screening strategies, while numerous studies are ongoing to develop specific pharmacological approaches, especially an effective vaccine [20,68,146]. Behavioral and lifestyle modifications are the cornerstone of prevention in pregnant women (mainly personal hygiene and education), followed by prenatal screening protocols. Recent advances in pharmaceutical treatment and vaccine development have shown promising, potentially life-changing results [68,147,148].

### 7.1. Promotion of Hygiene and Education

Education and hygienic practices remain the basis of cCMV prevention, given that approved pharmacologic interventions are currently lacking [149,150,151]. Pregnant women are advised not to come into contact with saliva and urine from young children, to avoid sharing utensils or food with them, and wash their hands regularly. Although adherence can be difficult, especially for mothers of young children, the aforementioned measures are highly effective when proper counseling is provided.

Studies demonstrated that healthcare provider-led counseling and educational tools—such as short videos or films used during prenatal care—can significantly enhance pregnant women’s awareness and adherence to preventive hygiene measures [152,153,154,155]. In an Italian study, targeted counseling of seronegative pregnant women significantly reduced the incidence of maternal infection, from 7.6–1.2% (80% reduction) [155]. In the United Kingdom, a digital intervention improved knowledge and reduced risky behaviors without increasing maternal anxiety [156]. The active involvement of healthcare professionals and the reinforcement of adherence are critical.

A semi-systematic review [157] highlighted that global awareness of CMV remains low. Likewise, surveys among healthcare professionals revealed significant gaps in the provision of CMV-related information. The Centers for Disease Control and Prevention (CDC) and other International Public Health Authorities, particularly the French High Council of Public Health, the American College of Obstetricians and Gynecologists (ACOG), the Society of Obstetricians and Gynaecologists of Canada and the ECCI recommend universal hygiene education during pregnancy as a primary strategy to reduce the burden of cCMV infection [18,20,68,158,159,160].

### 7.2. Prenatal Screening and CMV Testing

Universal prenatal screening for CMV is not currently recommended by major health organizations such as ACOG and the CDC, due to diagnostic limitations and the absence of approved interventions that reliably reduce fetal transmission [18,68]. False-positive IgM results and the possibility of viral reactivation in seropositive women reduce the clinical utility of generalized screening. Current guidelines advise that CMV serological testing should be carried out as early as possible during the first trimester of pregnancy. For women who test seronegative, it is recommended to repeat screening every four weeks until approximately 14 to 16 weeks of gestation. After 16 weeks, routine CMV testing is generally not advised unless ultrasound findings raise suspicion of CMV-related abnormalities [18,20].

Nevertheless, voluntary serologic screening is implemented in some European countries (e.g., Italy, Spain) aiming for early identification of primary infection and close clinical monitoring [161].

### 7.3. CMV-Specific Hyperimmune Globulin (CMV-HIG)

CMV-specific hyperimmune globulin (CMV-HIG) has been widely investigated as a form of passive immunoprophylaxis in pregnant women experiencing primary CMV infection, with the goal of decreasing the risk of intrauterine transmission. Early observational studies suggested a decrease in vertical transmission and symptomatic congenital disease [98], which stimulated considerable research interest. However, subsequent randomized controlled trials [66,98,162] and meta-analyses [163] revealed inconsistent and often statistically non-significant findings.

A phase II trial showed that monthly administration of 100 IU/kg CMV-HIG did not significantly reduce congenital infection compared to placebo and was associated with an increased rate of obstetric complications [161]. This prompted organizations like the Royal College of Obstetricians and Gynaecologists (RCOG) to advise against routine use of CMV-HIG [19]. A recent phase III multicenter trial [66] was prematurely discontinued due to lack of efficacy and the occurrence of serious adverse events (e.g., anaphylaxis).

Nevertheless, recent long-term observational studies [164,165] and large-scale systematic reviews and meta-analyses [163] suggested that, in selected cases of very early primary infection, CMV-HIG may reduce the risk of fetal transmission—especially when administered early and repeatedly [166]. According to current international guidelines, CMV-HIG is no longer recommended as a routine prophylactic therapy but may be considered in select cases of confirmed early primary infection. In summary, its use remains investigational and off-label, pending further evidence [16,19].

### 7.4. Antiviral Therapy for Managing cCMV

Recently, there has been an escalating interest in the prophylactic use of antivirals during pregnancy after primary CMV infection, in an attempt to lower the maternal viral load and inhibit vertical transmission. Even though effective antivirals, such as ganciclovir and foscarnet, show powerful anti-CMV activity, they are not used in pregnancy due to their known toxicity [167].

Valacyclovir, a prodrug of acyclovir, showed a better safety profile. Randomized controlled trials [168] and individual patient data (IPD) meta-analyses [10,169] demonstrated that its administration after confirmed primary infection during the first trimester is associated with a significant reduction in transplacental transmission. Similar findings were reported in multicenter studies and phase II trials investigating in-utero antiviral therapy [10]. A randomized study showed that daily administration of 8 g of valacyclovir from the first trimester reduced the risk of cCMV by up to 70% [168]. Particularly, the rate of fetal CMV infection was 11% in the valaciclovir group compared to 30% in the placebo group, indicating a 63% relative reduction in transmission. Specifically in the first trimester among the infected women, the transmission rate was 11% with valaciclovir vs. 48% with placebo, a 77% relative reduction. Likewise, other observational data confirmed reduced rates of vertical transmission, 61% relative reduction in the diagnosis of cCMV infection at the time of amniocentesis among women treated with valaciclovir compared to those who were not [20]. Reported adverse effects are generally mild and include nausea and transient renal impairment, while pharmacokinetic studies of maternal-fetal exposure indicate a favorable safety profile [168,169,170].

When compared to CMV-HIG, valacyclovir appears superior in terms of both efficacy and cost-effectiveness [20,170,171,172]. It is emerging as a promising alternative, particularly for women with documented primary infection in the first trimester [168]. At the moment, ACOG does not recommend its use outside of clinical studies [18]; however, recent European guidelines [20] support its inclusion in targeted clinical protocols. Valacyclovir might prove to be a useful preventive strategy, should its efficacy be confirmed in future large-scale trials.

### 7.5. Vaccines Against Human CMV Under Development

The development of a vaccine against human CMV has long been considered a public health priority, as congenital infection is associated with serious neonatal disabilities [173]. Early subunit vaccines such as gB/MF59 demonstrated modest efficacy (~50%) in preventing primary CMV infection in young women, motivating a shift toward more advanced vaccine platforms [174].

The mRNA-1647 vaccine (Moderna), which combines antigens for gB and the pentameric complex, induces strong neutralizing and T-cell immune responses and is currently undergoing phase III clinical trials [174]. If proven effective, it may be integrated into adolescent or preconception immunization programs, offering protection during the reproductive years [175,176]. Despite CMV’s immunological challenges, technological advances—particularly mRNA-based vaccines—offer promising new avenues for preventing congenital infection and reducing the global disease burden [65,177].

Given the substantial healthcare burden associated with CMV infections, the development of an effective and safe vaccine remains a critical public health objective. As early as 2000, the U.S. Institute of Medicine identified a CMV vaccine as a top priority among needed medical interventions, highlighting its potential to prevent considerable clinical and economic consequences. In fact, it is estimated that such a vaccine could lead to annual savings of approximately USD4 billion in the United States, primarily by reducing CMV-related complications in transplant recipients and congenital infections [178]. Challenges include the virus’ complexity, immune evasion capacity, and the need to trigger both neutralizing and non-neutralizing immune responses in susceptible individuals.

Vaccine development is focusing on two key antigens: glycoprotein B (gB), necessary for viral entry and a target of neutralizing antibodies, and the pentameric complex (gH/gL/UL128–131A), which evoke strong immune responses in epithelial and dendritic cells. Secondary antigens, such as IE1/IE2 and pp65, primarily stimulate T-cell immune responses [176,179,180,181].

Immunologically, protection against congenital infection appears to require a combination of high titers of neutralizing antibodies targeting both gB and the pentameric complex, as well as robust Th1-type T-cell responses. In contrast, immune responses targeting only pp65 are inadequate [182]. Non-neutralizing IgG functions, especially via Fc receptor-mediated mechanisms, were also associated with a reduced risk of vertical transmission [183].

#### 7.5.1. Categories of Candidate Vaccines

gB-Based Subunit Vaccines: One of the earliest and most studied gB-based formulations is the gB/MF59 subunit vaccine, which combines recombinant gB protein with the MF59 adjuvant. In a phase II randomized controlled trial, this vaccine demonstrated a 50% reduction in primary HCMV infection among seronegative women, making it the first to show partial efficacy in humans [184]. In a phase II RCT of CMV gB/MF59, local reactions were more frequent with vaccine than placebo (92% vs. 64%). Systemic reactions were also more common (82% vs. 67%), and fever > 38 °C occurred in 9% vs. 3%. No vaccine-related SAEs were reported. Unsolicited AEs occurred in 72% vs. 66% (NS), with higher rates of gastrointestinal (22% vs. 11%) and nervous system disorders (15% vs. 8%), but no significantly overrepresented AE type; there were 30 SAEs in 24 participants (none vaccine-related) [184]. A prior study found arthralgia (6% vs. 1%), chills (8% vs. 1%), and myalgia (first dose: 16% vs. 6%; third dose: 16% vs. 3%) occurred more often after vaccination, mostly mild and <1 day in duration. Local reactions were more frequent with vaccine, with severe pain (3%) and erythema (2%) after the third dose; other severe local reactions were ≤1%. No significant differences were seen in overall, moderate-or-greater, or serious AE rates (including in infants), though possibly vaccine-related AEs occurred more often with vaccine (7% vs. 2%) [185]. The immunological basis of protection appears to be linked not only to neutralizing antibodies but also to antibodies that inhibit cell-to-cell spread, a mechanism thought to play a critical role in controlling CMV dissemination in tissues [186].

Despite these promising findings, variability in vaccine-induced responses is a concern. Jenks et al. [187] reported that antibody binding to native gB—rather than to denatured or recombinant forms—correlates strongly with vaccine efficacy, suggesting that conformational epitopes are essential for protective immunity. This insight has prompted a shift toward next-generation approaches aimed at preserving native gB structure.

A major advance in this field is the development of nucleoside-modified mRNA vaccines encoding full-length gB, which elicit broader and more durable antibody responses than the protein/adjuvant formulation. In a comparative study, Nelson et al. [180] demonstrated that the mRNA platform induced potent binding and neutralizing titers, with superior persistence over time, reflecting enhanced immunogenicity and memory B-cell responses.

The relevance of gB-based immunity extends beyond prophylaxis in healthy individuals. In a cohort of seronegative transplant recipients, gB/MF59 vaccination prior to transplantation resulted in detectable neutralizing antibodies early post-transplantation, indicating potential for pre-emptive immunization strategies in high-risk populations [188].

Nonetheless, challenges remain. The gB/MF59 vaccine failed to achieve sterilizing immunity, and it elicited limited titers against fibroblast-independent viral entry pathways, suggesting the need for multivalent formulations or inclusion of other viral antigens such as the pentameric complex [180,187]. Major clinical limitations include (i) incomplete, context-dependent protection (strong in postpartum women but less compelling in adolescents), (ii) uncertain durability and immune correlates, (iii) limited strain/epitope coverage with a single-antigen gB strategy, and (iv) insufficient data on congenital CMV prevention due to small sample sizes and lack of power for neonatal endpoints [181,185].

In conclusion, gB-based vaccines have demonstrated proof-of-concept efficacy and immunogenicity, particularly in combination with adjuvants or advanced platforms like mRNA. However, optimization of antigen design to preserve native epitopes, broad neutralization across cell types, and synergy with other antigens will be essential steps toward a highly effective CMV vaccine [176,180,187].

mRNA Vaccines: The most promising platform to date is mRNA-1647 (Moderna), which incorporates both gB and pentameric complex antigens. It has demonstrated superior immunogenicity compared to gB/MF59 in phase I/II trials [189].

The mRNA-1647 vaccine, developed by Moderna, uses a non-replicating mRNA platform encapsulated in lipid nanoparticles (LNPs) to deliver instructions to host cells for the in vivo expression of key CMV antigens. It contains six mRNAs, encoding: one full-length membrane-bound gB—crucial for viral entry and fusion with host cells (especially fibroblasts), and five components of the pentameric complex (gH/gL/UL128/UL130/UL131A)—essential for CMV entry into epithelial and endothelial cells, the key cell types in maternal–fetal transmission. These mRNA sequences prompt host cells to synthesize antigens in their native structures, which in turn triggers both antibody-mediated and cell-mediated immune responses. Specifically, they induce high titers of neutralizing antibodies (nAbs) targeting gB and the pentameric complex, robust CD4^+^ and CD8^+^ T-cell responses, and the expansion of memory B cells that produce CMV-specific IgG. The pentamer-specific IgG responses dominate and are key to neutralizing CMV in epithelial cells, the primary entry route during congenital infection [176,189].

Clinical development of the CMV mRNA vaccine has progressed through multiple phases, demonstrating promising results. In Phase I trials (2017–2020), the vaccine showed a favorable safety profile and elicited strong immune responses in both CMV-seronegative and seropositive adults. Neutralizing antibody levels peaked around the seventh month and remained elevated up to one year. Reported side effects were mostly mild to moderate, with fatigue, headache, and muscle pain being the most common [190]. Injection-site pain occurred in 67–85% (phase B) and 90–100% (phase C), with higher rates at 180–300 μg and peaking after dose 1. Systemic ARs were more common and severe in seropositive participants, ranging from 38–75% (phase B) to 79–100% (phase C), increasing with dose. No deaths, treatment-related SAEs, or AESIs occurred. Unsolicited AE rates were similar between groups, with rare treatment-related MAAEs (e.g., urticaria, dizziness, arthralgia, gastroenteritis). All SAEs were unrelated; no withdrawals were due to AEs [190]. In a phase II trial of 315 adults, solicited ARs after dose 1 occurred in 84–91% of seronegative and 94% of seropositive mRNA-1647 recipients vs. ~55–59% with placebo. Pain (73–89% vs. ~19%) and fatigue (29–72% vs. ~26–30%) were most common, with similar patterns after doses 2–3 [168]. During Phase II, the 100 µg dose was identified as the most effective, producing strong antibody responses across different serostatus groups while maintaining acceptable safety [168]. While the vaccine elicits strong neutralizing antibody responses, key limitations remain. There is no direct evidence for preventing congenital CMV (no vertical transmission endpoints), undefined correlates of protection, unknown durability beyond 12–18 months and need for boosters, common reactogenicity, and limited generalizability to the studied age and health groups [168,190]. The Phase III trial, which is currently ongoing, is enrolling 7454 women of reproductive age. Preliminary data indicate that the vaccine continues to induce robust immune responses with good tolerability. Final results, expected by 2026, will determine its effectiveness in preventing cCMV infection [167,189].

Vectored Vaccines platforms: Vectored vaccine platforms offer an innovative approach to CMV prevention by delivering antigen-encoding genes into host cells, enabling endogenous expression and presentation of immunodominant viral proteins. This strategy stimulates both cellular and humoral immunity, a crucial feature for combating CMV, which requires strong CD8+ T -responses in addition to neutralizing antibodies [191].

Among the most studied vectors is the Modified Vaccinia Ankara (MVA)-based Triplex vaccine, which encodes pp65, IE1-exon4, and IE2-exon5. The Triplex vaccine showed potent T-cell immunogenicity in transplant recipients, boosting CMV-specific CD8+ and CD4+ T cells, and inducing adaptive NK cells, without significant safety concerns [192,193]. The Triplex MVA-vectored vaccine (pp65, IE1, IE2) in CMV-seropositive HCT recipients showed no serious or grade 3–4 vaccine-related AEs, no nonrelapse deaths within 100 days, and similar severe acute GVHD rates to placebo (HR 1.1; 95% CI 0.53–2.4). Limitations include a narrow target population, exclusion of key high-risk groups (e.g., pregnant women, children, HIV-positive individuals), a primary endpoint limited to post-letermovir transplant reactivation rather than broader CMV prevention, and a prolonged projected accrual period likely to delay definitive efficacy findings [193,194].

Another key candidate is Merck’s V160, a replication-defective CMV vaccine that restores the pentameric complex and mimics natural infection without productive viral replication. In Phase 2b trials, V160 induced robust T-cell responses and neutralizing antibodies, comparable to natural infection, although efficacy in preventing primary CMV infection was moderate (~42%) [195]. In phase 1′ CRT injection-site, AEs occurred in 58–100% (seropositive) and 67–100% (seronegative), most often pain with IM and erythema with ID administration; most were mild–moderate. Systemic AEs occurred in 64–91% and 67–100%, respectively, vs. 50–58% and 56–100% with placebo, with mainly fatigue and headache. Severe cases occurred in four seropositive and seven seronegative participants; four discontinued due to AEs. Fever ≥38 °C occurred in 4% and 9%, with no ≥40 °Cb [196]. In a separate cohort, AEs occurred in 83% of seropositive and all seronegative V160 recipients vs. 67% and 33% with placebo [197]. While V160 was well tolerated, its use is limited by the lack of published efficacy data for preventing primary infection or congenital transmission, undefined correlates of protection, unknown durability of immunity, and untested performance in wider or higher-risk populations [196,197].

HB-101, created with non-replicating lymphocytic choriomeningitis virus vectors, encodes the CMV gB and pp65 antigens. In Phase 1–2 studies, HB-101 elicited strong pp65-specific CD8+ T-cell responses and neutralizing antibodies, with early signs of efficacy in CMV-seronegative kidney transplant candidates [198,199]. In a phase I trial, solicited local/systemic symptoms were mild–moderate, with 96.5% resolving within 8 days (max 10 days) [198]. Injection-site pain was the most common local AE; malaise, fatigue, and myalgia were the most common systemic AEs. Local/systemic AE rates were higher in the high-dose group vs. placebo, with injection-site pain increasing after each dose but no cumulative systemic AE effect. No abnormal labs or vital signs were observed.

While used primarily in transplant recipients, some are under investigation for use in pregnancy prophylaxis. These vaccines often encode pp65 or IE1 to activate T-cell responses, although their immunogenicity remains modest in the absence of strong adjuvants [200]. Vectored vaccines against CMV demonstrated promising immunogenicity and acceptable safety profiles in clinical trials [191]. The vaccine’s clinical applicability is limited by the absence of efficacy data for preventing congenital CMV or CMV disease in transplant recipients, undefined optimal dosing and immune durability, data restricted to small healthy adult cohorts, and lack of established correlates of protection, warranting further assessment in larger, more diverse phase 2/3 trials [198]. Nanoparticle, computationally designed, or virus-like particle (VLP) vaccines—VLP vaccines represent an innovative and promising platform for the prevention of viral infections, offering structural mimicry of native virions without containing infectious genetic material [201,202]. Their mechanism of action is based on the dense, repetitive, and multivalent display of viral antigens, which facilitates efficient recognition by pattern recognition receptors and uptake by antigen-presenting cells (APCs), such as dendritic cells. Once internalized, VLPs undergo processing and antigen presentation via MHC class I and II pathways, leading to the activation of CD4^+^ T helper cells, cytotoxic CD8^+^ T lymphocytes, and robust B cell-mediated neutralizing antibody responses. This architecture enhances immunogenicity while minimizing safety concerns, making VLPs suitable for both naive and immunocompromised populations [203].

A recent first-in-human trial demonstrated the clinical potential of a VLP-based vaccine for human CMV; Langley et al. [204] assessed an enveloped virus-like particle (eVLP) vaccine that expressed a modified version of the CMV gB, combined with an aluminum hydroxide (alum) adjuvant and tested in healthy adults who were seronegative for CMV. The vaccine was well tolerated and induced strong immunogenicity, with dose-dependent increases in binding and neutralizing antibody titers. Notably, 100% of participants receiving the highest dose (2 μg of gB + alum) developed neutralizing activity in fibroblast-based assays, and 31% exhibited neutralizing responses in epithelial cell assays—two distinct CMV entry pathways. Additionally, approximately 24% of high-dose recipients generated antibodies targeting the AD-2 epitope of gB, a known correlation of broad neutralization. In phase 1, eVLP-gB + alum caused only expected injection-site pain and transient systemic symptoms, most commonly pain (local) and headache (systemic). No SAEs or withdrawals occurred [205]. These findings not only validate the safety and immunogenic potential of CMV VLPs but also support their further clinical development, particularly in maternal immunization strategies for the prevention of cCMV transmission [204].

Beyond CMV, the VLP platform has demonstrated broad applicability for emerging and re-emerging viral threats. Laxmi et al. [203] highlighted the modular nature of VLPs, which can be engineered to incorporate multiple antigens or toll-like receptor (TLR) ligands, enhancing humoral and cellular responses. Their physicochemical stability, amenability to large-scale production, and favorable safety further underscore their relevance in rapid pandemic response settings. By integrating the potent immunogenicity of whole-virus vaccines with the enhanced safety of subunit formulations, VLP-based vaccines represent a compelling platform for next-generation vaccine design. Despite its advantages, clinical applicability is constrained by the lack of long-term follow up, undefined correlates of protection, and evaluation limited to a narrow healthy adult cohort, underscoring the need for broader, advanced trials to determine efficacy, durability, and safety in diverse at-risk populations [203]. Table 1 presents a summary of key data on the main candidate vaccines against CMV, including the platform, the company/candidate name, the phase of clinical development, main findings, and whether data on use in pregnant individuals are available.

#### 7.5.2. Clinical Efficacy and Safety

The evaluation of clinical efficacy and safety of CMV vaccines is particularly complex due to the heterogeneous nature of target populations (e.g., pregnant women, seronegative individuals, transplant recipients), the partial protective immunity conferred by natural infection, and the lack of well-defined correlates of protection. In a recent systematic review of randomized controlled trials, Chiavarini et al. [191] assessed the safety, immunogenicity, and efficacy of CMV vaccines, including subunit (e.g., gB/MF59), DNA-based, and viral vector platforms. Nine RCTs, involving both healthy volunteers and special populations (e.g., transplant candidates), were identified, and it was concluded that CMV vaccines were generally well-tolerated, with no significant increase in severe adverse events compared to placebo. Most local and systemic adverse reactions were mild to moderate in intensity and transient in duration. With respect to clinical efficacy, the review confirmed that the gB/MF59 subunit vaccine demonstrated approximately 50% efficacy in preventing primary CMV infection among seronegative women, consistent with earlier findings. However, other vaccine platforms, including DNA vaccines and viral vectors, showed variable or limited efficacy, particularly in transplant settings. The authors highlight the need for longer follow-up, standardized definitions of infection, and improved assessment of congenital transmission outcomes to enable robust comparisons across vaccine candidates [191].

From a regulatory and scientific perspective, Krause and Roberts [207] underscore the methodological difficulties in conducting CMV vaccine trials. Among these are the high background seroprevalence, the lack of sterilizing immunity even after natural infection, and the absence of universally accepted immune correlations. These challenges complicate trial design and regulatory approval processes. They emphasize the clinical endpoints such as the following: (i) prevention of primary maternal infection defined by seroconversion to non-vaccine CMV antigens and/or detection of incident CMV DNAemia/virumia following vaccination, (ii) reduction of cCMV transmission confirmed in the neonate by CMV PCR or culture from saliva/urine within ≤21 days postpartum, and (iii) mitigation of clinically significant neonatal outcomes, including symptomatic cCMV, sensorineural hearing loss assessed by serial auditory brainstem response, and neurodevelopmental impairment measured via standardized scales during early life [174,185,208,209,210]. Secondary maternal virologic/immunologic parameters, peak and AUC of CMV DNA load, neutralizing antibody breadth to gB and pentameric complex, IgG avidity, and CMV-specific T-cell responses, serve as correlations of protection [174,210]. In infection cases, placental assessment can be of paramount importance in the evaluation of the efficacy of CMV vaccination. It can provide mechanistic insights: virologic assays (placental CMV DNA load via qPCR; RNA in situ hybridization), histopathology (villitis, intervillositis, fibrinoid necrosis), and immunopathology (cytokine signatures such as TNF-α and IL-6 upregulation) quantify tissue involvement and relate to fetal disease risk [209,210,211,212]. In a guinea pig cytomegalovirus (GPCMV) challenge model, preconception immunization with a GP83-deletion vaccine significantly reduced maternal viremia (DNAemia) and conferred protection against pup mortality associated with congenital GPCMV infection, compared with unvaccinated controls [213]. Vaccination was also associated with decreased placental viral burden, likely secondary to the reduction in maternal DNAemia. These measures together capture both sterilizing immunity and disease-modifying effects [214,215,216]. Overall, the authors advocate for the use of harmonized serological and virological assays, consistent monitoring protocols, and well-defined high-risk populations in efficacy studies. They also highlight the importance of post-licensure surveillance systems to detect rare adverse events and assess long-term effectiveness in real-world settings [207].

Several vaccine candidates demonstrated promising safety and immunogenicity profiles, including adjuvanted gB subunit vaccines and DNA-based constructs targeting gB and pp65 [191]. However, long-term protection remains elusive with gB/MF59, prompting a shift toward multivalent vaccine strategies, especially those incorporating the pentameric complex to broaden neutralizing antibody responses. Notably, the mRNA-1647 vaccine, a multicomponent mRNA-based candidate encoding both gB and the pentamer complex, demonstrated strong antibody responses and mild side effects in early-phase trials. It is presently the only CMV vaccine advancing through phase III clinical trials [167,189]. Despite these advances, no CMV vaccine has yet received regulatory approval, and their efficacy in preventing congenital transmission remains under active investigation.

In conclusion, current evidence supports the favorable safety profile of CMV vaccine candidates and moderate clinical efficacy, particularly in seronegative women of childbearing age. Nonetheless, the regulatory pathway remains challenging, and future trials must incorporate better endpoints, immunological markers, and population-specific strategies to accelerate vaccine licensure and deployment [191,207].

#### 7.5.3. Barriers in Development

The development of vaccines to prevent cCMV infection faces several complex barriers, including the virus’ extensive genetic diversity, its capacity to establish lifelong latency, sophisticated immune evasion strategies that undermine host defenses, and the unresolved challenge of identifying the most appropriate population for targeted immunization.

Current evidence positions CMV vaccination primarily for CMV-seronegative women of child-bearing age, with the goal of preventing primary maternal infection during pregnancy, and for solid-organ or hematopoietic stem cell transplant recipients to reduce the risk of CMV disease or reactivation. Other proposed target groups include healthcare and childcare workers with high occupational exposure [181,188,191,217,218]. In high-seroprevalence settings, Baraniak et al. [188], highlight that a substantial proportion of congenital CMV results from non-primary infection in seropositive women, indicating that vaccine strategies must also address reinfection and reactivation.

Although no formal contraindications have yet been defined from trial data, most studies have excluded pregnant women, immunocompromised patients outside target transplant populations, and those with severe chronic illnesses, and live-attenuated candidates such as V160 are considered precautionarily contraindicated in immunosuppressed individuals and during pregnancy until safety is established [181,188,191,217,218]. Special populations of interest include immunosuppressed patients, particularly transplant recipients, and HIV-positive individuals, both of whom have elevated CMV risk but have been largely excluded from vaccine trials, leaving critical evidence gaps. Chiavarini et al. [191] specifically highlight HIV-positive women of child-bearing age as a priority group for future evaluation given their increased risk of congenital CMV transmission. Collectively, all reviews stress that no CMV vaccine has yet been proven safe or effective during pregnancy, in infants, or in children.

Prevention of cCMV infection in seronegative women may be achievable through vaccination strategies capable of inducing robust humoral and cellular immune responses against highly immunogenic viral components implicated in CMV pathogenesis. Vaccines targeting glycoprotein B (gB), the pentameric complex, and pp65 showed promise in significant reduction of the risk of CMV transmission. In contrast, preventing cCMV infection in seropositive pregnant women will likely require targeted interventions addressing the mechanisms involved in non-primary CMV infections, such as viral reactivation or reinfection [219]. mRNA technology currently stands out as the most promising approach for CMV vaccine development. If mRNA-1647 proves effective, it may represent a turning point in public health and reduce the global burden of perinatal complications [189].

Nonetheless, the availability of effective vaccines alone may not fully resolve the challenges associated with cCMV prevention. A key uncertainty still lies in how long the immunity provided by the vaccine will last. Establishing long-term protection would be essential to support a universal immunization strategy, potentially including the vaccination of preadolescent girls—similar to current recommendations for the human papillomavirus vaccine [220]. Vaccination before conception seems to be the most effective approach. The main target group for vaccination is seronegative women of childbearing age, aiming to prevent primary maternal CMV infection during pregnancy [181], but further data on long-term efficacy and cost-effectiveness are needed [219]. A recent study by Ge et al. [221], reported that CMV vaccination for women prior to marriage was a cost-effective strategy to control cCMV infection in China. These findings can inform public health policies and guide future research on optimizing CMV vaccination strategies.

## 8. Conclusions

Congenital CMV infection constitutes a major global public health concern, with potentially severe short- and long-term consequences for affected neonates, their families, and society as a whole. Neurodevelopmental impairments, SNHL, and other disabling outcomes associated with cCMV significantly impact healthcare systems and diminish patients’ quality of life. Early diagnosis through targeted prenatal and neonatal screening is therefore critical for timely intervention and improved clinical outcomes.

Despite these efforts, prevention remains the most effective strategy for mitigating the impact of cCMV, with vaccination emerging as the most promising approach. Continued investment in the research and development of a safe and effective CMV vaccine is essential to reduce both the incidence and the socioeconomic burden of the disease. Incorporating such a vaccine into national immunization programs could play a pivotal role in reducing transmission, particularly through routine vaccination of adolescents or women of reproductive age.

## Figures and Tables

**Table 1:** Overview of CMV vaccine candidates by platform.

Vaccine Platform	Key Candidates	Trial Phase	Key Findings	Pregnancy-Specific Data
**gB-Based Subunit Vaccines**	gB/MF59 [184]	Phase II (completed)	~50% efficacy in seronegative women; antibody responses to native gB important; limited cell-to-cell spread neutralization	No approval for use in pregnancy; limited safety data available
**mRNA Vaccines**	mRNA-1647 [167]	Phase III (ongoing)	Encodes full-length gB + pentamer complex; strong neutralizing Ab qne T-cell responses; long-lasting immunity	Phase III includes women of childbearing age; results expected by 2026. Not currently approved for pregnant women
**Viral Vectored Vaccines**	Triplex (MVA) [197], V160 [195], HB-101 [198,199]	Phase I–II	Strong T-cell responses (esp. pp65, IE1); V160 showed ~42% efficacy; well tolerated	Mostly studied in transplant recipients; not yet approved or sufficiently tested in pregnant populations
**Virus-Like Particle (VLP) Vaccines**	eVLP-gB + alum [204]	Phase I (first-in-human)	Well tolerated, potent antibody titers including AD-2 response; 100% neutralization in fibroblasts at high dose	Suggested for maternal immunization, but no current pregnancy-specific trials or approval
**Next-Gen/Computational Platforms**	Multivalent VLPs, epitope-optimized constructs [206]	Preclinical	Designed to enhance breadth/durability; TLR ligand integration; scalable and stable	Preclinical only; not yet tested in humans, including pregnant women

## Data Availability

Data are contained within the article.
